# Investigating student interactions with tutorial dialogues in EER-Tutor

**DOI:** 10.1186/s41039-015-0013-1

**Published:** 2015-07-28

**Authors:** Myse Elmadani, Antonija Mitrovic, Amali Weerasinghe, Kourosh Neshatian

**Affiliations:** 1 Intelligent Computer Tutoring Group, University of Canterbury, Christchurch, New Zealand; 2 School of Computer Science, University of Adelaide, Adelaide, Australia; 3 Department of Computer Science and Software Engineering, University of Canterbury, Christchurch, New Zealand

**Keywords:** Tutorial dialogues, Constraint-based intelligent tutoring system, Eye tracking, Data mining

## Abstract

Eye-movement tracking and student-system interaction logs provide different types of information which can be used as a potential source of real-time adaptation in learning environments. By analysing student interactions with an intelligent tutoring system (ITS), we can identify sub-optimal behaviour such as not paying attention to important interface components. On the basis of such findings, ITSs can be enhanced to be proactive, rather than reactive, to users’ actions. Tutorial dialogues are one of the teaching strategies used in ITSs which has been shown empirically to significantly improve learning. Enhanced entity-relationship (EER)-Tutor is a constraint-based ITS that teaches conceptual database design. This paper presents the preliminary results of a project that investigates how students interact with the tutorial dialogues in EER-Tutor using both eye-gaze data and student-system interaction logs. Our findings indicate that advanced students are selective of the interface areas they visually focus on, whereas novices waste time by paying attention to interface areas that are inappropriate for the task at hand. Novices are also unaware that they require help with the tutorial dialogues. Furthermore, we have demonstrated that the student’s prior knowledge, the problem complexity and the percentage of the dialogue’s prompts that are answered correctly are factors that can be used to predict future errors. The findings from our study can be used to further enhance EER-Tutor in order to support learning better, including real-time classification of students into novices and advanced students in order to adapt system feedback and interventions.

## Background

Despite the proven effectiveness of intelligent tutoring systems (ITSs), studies indicate that some students only gain shallow knowledge which they then have difficulty applying to new and different problems (Aleven et al. 1999). One of the ways to overcome this shallow learning problem is to engage in metacognitive activities such as self-explanation and reflection (Chi et al. 1989). Self-explanation is a constructive activity during which a person tries to make sense of new information by explaining it to him/herself (Chi 2000). This results in the revision of his/her knowledge for future application.

One instructional strategy that supports self-explanation and reflection is tutorial dialogues. Tutorial dialogues have been used in a number of ITSs in order to encourage deep learning. In some systems, such as Why2-Atlas (Vanlehn et al. 2002) and Auto Tutor (Graesser et al., 2003), tutorial dialogues are used as the main learning activity. In contrast, systems like Geometry Explanation Tutor (Aleven et al. 2004) and KERMIT-SE (Weerasinge and Mitrovic 2005) use problem-solving as the primary learning activity, while tutorial dialogues provide additional support. The Geometry Explanation Tutor allows students to give natural language explanations about their problem-solving steps. KERMIT-SE asks students to justify only problem-solving decisions that led to incorrect solutions. Tutorial dialogues have been evaluated empirically and shown to significantly improve learning (Olney et al. 2010; Weerasinghe et al. 2011).

This paper outlines work in progress that investigates how students interact with tutorial dialogues using two information sources: eye-gaze data and student-system interaction logs. Our goal is to identify ineffective student behaviour, which would enable us to enhance the ITS to support learning better. The results of such investigations could therefore enable proactive rather than reactive instructional actions taken by an ITS.

The project will give us a better understanding of how different students interact with tutorial dialogues in an ITS. For example, we can investigate if the dialogue contents are being visually attended. One of the obvious factors behind students interacting differently with tutorial dialogues in an ITS is the amount of prior domain-specific knowledge that the student has. By dividing students into novice and advanced groups, we can therefore pinpoint group-specific behaviours. We believe that we can detect sub-optimal student behaviour from eye tracking and/or ITS logs in order to allow an ITS to intervene when needed and better guide students’ learning. We will get an indication about whether eye tracking gives us a more complete picture of students’ interactions and whether the cost of eye tracking can be justified. For example, we may be able to use time-based evidence to determine if a student is not paying much attention to the tutorial dialogue by looking at the time between the dialogue appearing and the student responding. However, this does not reveal situations in which the student is taking time to respond but is visually attending to irrelevant areas of the interface. A comparison of the accuracy of classifying students’ behaviour based on these different measures would be useful. Furthermore, enabling the ITS to predict the occurrence of errors would allow for a more personalised learning experience. Because we have already implemented adaptive tutorial dialogues in enhanced entity-relationship (EER)-Tutor (Weerasinghe et al. 2011), we will use this constraint-based ITS. EER-Tutor teaches database design using EER data modelling (Elmasri and Navathe 2007).

We present EER-Tutor in the following section and discuss related work on using eye-tracking data in the ‘Related eye-tracking research’ section. ‘Methods’ section outlines the study we carried out, followed by a discussion of results in the ‘Analysing EER-Tutor logs’ and ‘Analysing eye-tracking data’ sections. Finally, we present conclusions and future research plans in the ‘Conclusions and future work’.

### EER-Tutor

**Fig. 1:**
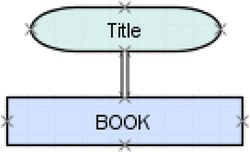
An example of a constraint violation

EER-Tutor (Zakharov et al. 2005) is a constraint-based ITS which provides a learning environment for students to practise conceptual database design using the EER data model. KERMIT (Suraweera and Mitrovic 2004) was an earlier version of this ITS which taught the basic version of the ER model. A *constraint* is an ordered pair (*C*_*r*_, *C*_*s*_) where *C*_*r*_ is the relevance condition and *C*_*s*_ is the satisfaction condition. A constraint-based ITS evaluates a submitted solution by checking it against constraints that are relevant for that solution. If the relevance condition is met, that constraint is applied and the satisfaction condition must also be met. Otherwise, the constraint is *violated*, indicating an error in the submitted solution. The student model tracks the constraint’s usage over time. One constraint in EER-Tutor, for instance, specifies that a single line must be used to connect an attribute to another construct. The solution in Fig. 1 is therefore incorrect as this constraint is violated.

**Fig. 2:**
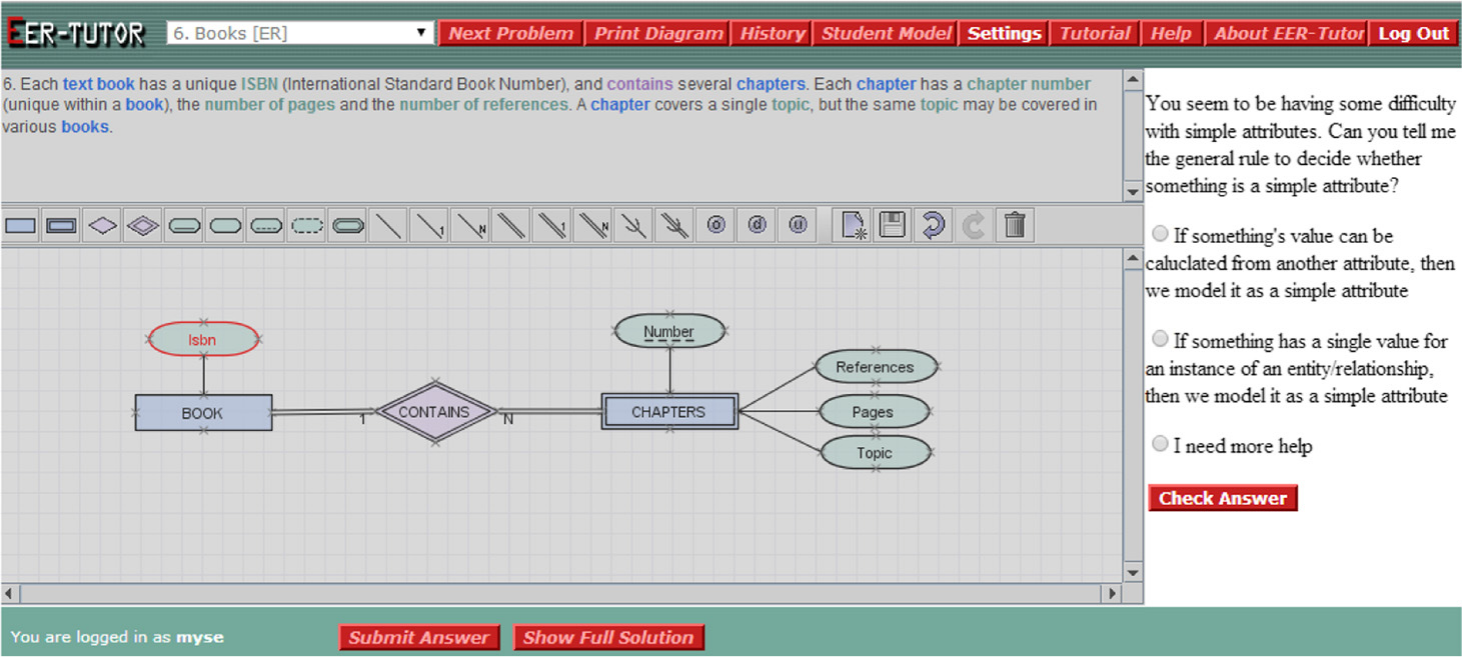
The EER-Tutor interface

The EER-Tutor interface in Fig. 2 shows the problem statement at the top, the toolbox containing the components of the EER model, the drawing area on the left and the feedback area on the right. Students create EER diagrams satisfying a given set of requirements which are checked for constraint violations on submission. EER-Tutor records detailed session information, including each student’s attempt at each problem and its outcome.

Tutorial dialogues have been implemented for KERMIT (Weerasinghe and Mitrovic, 2006) and later for EER-Tutor (Weerasinghe et al. 2008; Weerasinghe et al. 2011). The model for supporting adaptive dialogues is beyond the scope of the current paper, and we refer the interested reader to Weerasinghe et al. (2009) for details. Here, we present only a short explanation of the tutorial dialogues.

**Fig. 3:**
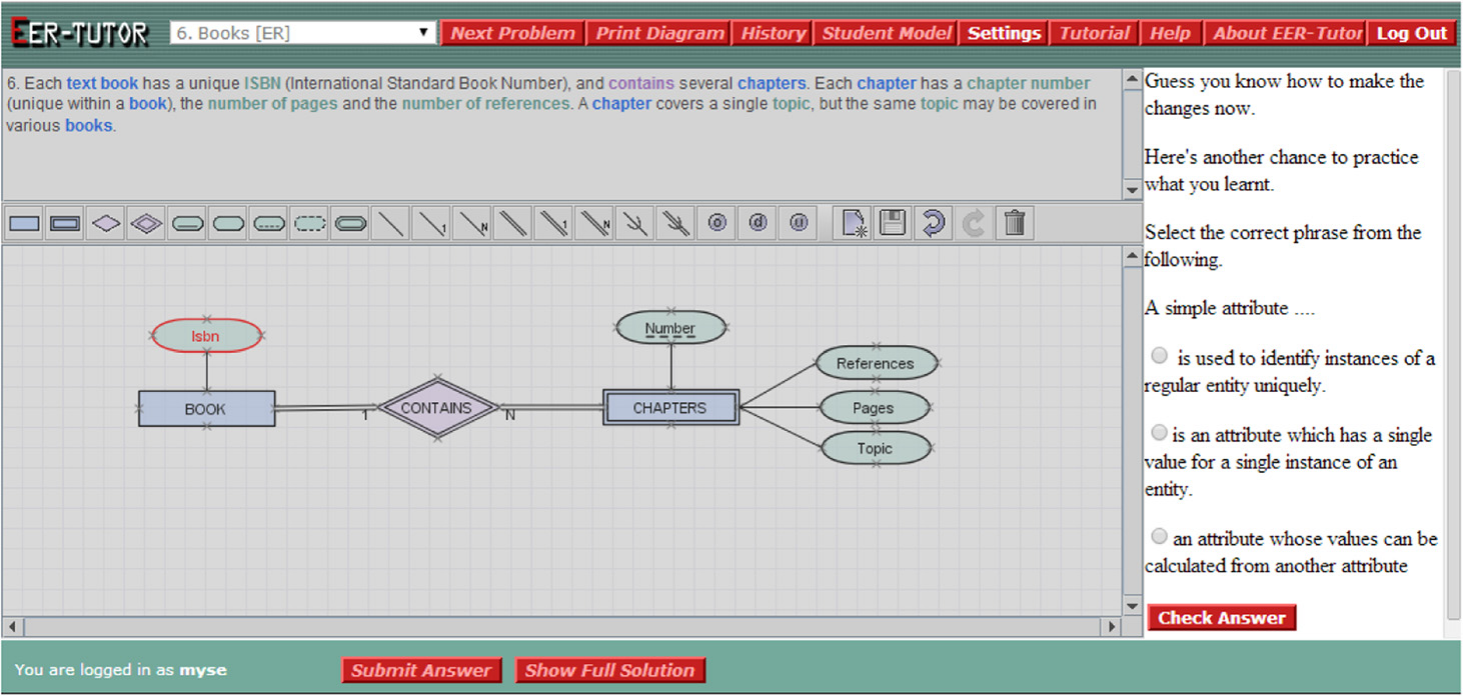
A conceptual reinforcement prompt

When a student makes one or more mistakes, s/he is presented with a tutorial dialogue selected based on the student model. The problem statement, toolbar and drawing area are disabled but visible for the duration of the dialogue, and the error is highlighted in red in the diagram. Each dialogue consists of several prompts and provides multiple possible answers to the student. Therefore, the student answers prompts by selecting an option s/he believes is correct or asks for additional help by selecting the “I need more help” option. The prompt types analysed are as follows:

- Conceptual (CO): discusses the domain concept with which the student is having difficulty independently of the problem context. This is shown in Fig. 2: the student has modelled ‘Isbn’ as a simple attribute instead of a key attribute so the prompt is asking about the basics of simple attributes. An incorrect answer to a conceptual prompt results in an additional, simpler conceptual prompt being given.
- Reflective (RE): aims to help students understand why their action is incorrect in the context of the current problem and therefore the prompt text refers to the elements of the problem. For the error in Fig. 2, this is: “Can you tell me why modelling Isbn as a simple attribute is incorrect?”
- Corrective action (CA): gives the student the opportunity to understand how to correct the error for this specific problem. For the error in Fig. 2, the CA prompt is asking the student to specify the best way to model the Isbn attribute and giving the different attribute types as options. Not all dialogues have this prompt type.
- Conceptual reinforcement (CR): allows the student to review the domain concept learned. For the error in Fig. 2, the CR prompt asks the student to choose the correct definition of a simple attribute from the given options as seen in Fig. 3. This is a problem-independent prompt.

In addition to multi-level dialogues, students are given single-level hints when they make basic syntax errors such as leaving diagram elements unconnected. We use prompts and dialogues from now on to refer only to multi-level dialogues unless otherwise specified.

### Related eye-tracking research

Eye tracking is concerned with determining the point of gaze of a person’s eyes. The human eye makes a series of rapid eye movements (saccades) then fixates on some point in a visual scene (a fixation) (Goldberg and Helfman 2010). In order to help with eye-tracking analysis, areas of interest (AOIs) can be set up that specify important regions in the user interface. AOIs are useful for tallying fixations and identifying scanning sequences and transitions, for example, Goldberg and Helfman (2010).

Eye tracking is used in interface usability studies, advertising as well as developmental psychology. As regards to ITSs, the use of eye tracking to increase student model bandwidth for educational purposes was first discussed in Gluck et al. (2000). Eye tracking was also used as a form of input (Wang et al. 2006), by allowing students to select a topic to study by simply looking at a portion of the screen for a pre-specified time or answer questions using eye movements. Other researchers used eye-tracking data to analyse how students interpret open learner models (Bull et al. 2007; Mathews et al. 2012).

We have categorised related studies under three categories: *eye tracking for user classification*, *eye tracking and attention/affective state* and *eye tracking and graphics/visualisations*. In the first category, eye tracking is used to directly augment the student model or understand different groups of students. We then outline the research work involving the use of eye tracking to understand or predict the students’ affective state, in particular to determine when students are struggling. A review of the research into using eye tracking to learn more about students’ behaviour when viewing visualisations and graphics follows, and a summary of the findings of the related work concludes this section.

#### Eye tracking for user classification

Conati and Merten (2007) carried out online assessment of students’ self-explanations using eye-tracking data and interface actions. Students used the Adaptive Coach for Exploration (ACE), an exploration-based learning environment that allows students to explore mathematical functions (Bunt et al. 2001). An empirical evaluation of a probabilistic user model including self-explanation found that gaze-tracking data improved model performance compared to using only time data as a predictor of self-explanation.

In other work, Kardan and Conati (2012) used users’ visual attention patterns only to assess their ability to learn with an interactive simulation. The simulation used shows how an algorithm for solving constraint satisfaction problems works. The authors also found that the changes in users’ attention patterns when moving to solving a more difficult problem can be used to classify students based on their performance. For example, high achievers increase the number of fixations on an AOI that should be used more in the harder problem.

The following studies specifically use expertise as the method of grouping students. In contrast to the studies above, where participants were all from the same course or year level at the university, participants were recruited for these studies from known expert and novice groups. An expert is a participant who has a number of years of experience in a particular domain for example, whereas a novice is a participant who is taking an introductory course.

Differences between novice and expert pilots were found using eye-tracking data gathered during simulated visual flight rules flight landings (Kasarskis et al. 2001). Experts had more frequent fixations on relevant areas but for shorter durations. The scan patterns of experts are also stronger and more defined, which means that they better maintain airspeed and good landing performance because the patterns are consistent and efficient.

Law et al. (2004) also used eye-gaze patterns to differentiate between novices and experts. Novice and expert surgeons performed a task on a computer-based laparoscopic surgery simulator. Expert surgeons were quicker and made fewer errors overall as expected. Novices had to fixate on the tool’s position and had varied behaviours, whereas experts could manipulate the tool while maintaining eye gaze on the target.

Jarodzka et al. (2010) similarly investigated the differences in strategies used by novices and experts and therefore the areas they fixate on. When asked to describe the locomotion patterns of swimming fish from a video, experts attended to task-relevant features more than novices and remained focussed on these areas. In addition, experts focussed on different features because they employed knowledge-based shortcuts unknown to novices.

While the next group of studies is still concerned with understanding different student groups, the context is specifically that of problem-solving tasks (not necessarily using an ITS).

Eivazi and Bednarik (2011) proposed the use of real-time tracking of users’ visual attention patterns to model users’ high-level cognitive states and performance. The rationale is that an intelligent system can monitor users and use eye-movement data to guide learning. Eye-movement features were calculated for each interval corresponding to an utterance coded to a cognitive trait such as planning while solving an eight-tile puzzle. A support vector machine-based classification was used to predict problem-solving cognition states such as planning as well as a user’s performance. Performance was accurately predicted: the high-performance group had a lower number of fixations but longer fixation durations than the low-performance group for example.

Tsai et al. (2012) used eye tracking to study students’ visual attention when predicting debris slide hazards in an image-based, multiple-choice science problem. Students attended more to the option they chose than the options they rejected and spent more time inspecting features relevant to the answer chosen than features that are irrelevant to it. Regarding successful problem solvers, the study found that they shift gaze from irrelevant to relevant features. This is in contrast to unsuccessful problem solvers who shift their gaze from relevant to irrelevant features and the problem statement.

#### Eye tracking and attention/affective state

Gluck et al. (2000) uses eye tracking to increase student model bandwidth. Students used the EPAL Algebra Tutor, an adaptation of the Worksheet tool in Algebra Tutor (Koedinger and Anderson 1998). The findings showed that students ignore bug messages and that some students also ignore algebraic expressions, so the tutor could draw their attention to these areas.

Wang et al. (2006) use eye tracking as both input and a source of information for adaptation in an emphatic software agent (ESA) teaching Biology. Topic selection for example is done through the student gazing at the relevant area for a predetermined amount of time. The agents themselves also adapt their behaviour in accordance with the student’s state as inferred by his/her eye movements, pupil dilation and changes in eye position. For instance, if the student is continually looking away from the screen, the agent will appear mildly angry and remind him/her to concentrate whereas fixating on an area will cause the agent to move there and display more detailed content. The preliminary usability study indicated that the students paid attention to explanations and content given by the agents.

#### Eye tracking and graphics/visualisations

The user’s knowledge affects which parts of the interface are visually attended, i.e. the user spends more time looking at task-relevant areas and the fixation duration increases during learning (Canham and Hegarty 2010). Canham and Hegarty also found that giving users less complex weather maps (containing no extraneous information) meant they performed better because only task-relevant information was displayed.

Bull et al. (2007) investigated how students explore different representations of their open learner models (OLMs) for the domain of C programming. The student’s preference for OLM visualisation did not affect the information s/he visually attended, but certain representations did encourage visual attention on information about how much domain knowledge s/he has. The representation of an OLM therefore needs to be considered in the context of its overall purpose. By focussing on showing the amount of knowledge students have for example, students are prompted to reflect on and become more aware of the gaps in their knowledge. Another factor that should be considered when designing OLMs is the visualisation complexity—more complex representations require more effort to understand and therefore result in a broader spread of visual attention.

Conati et al. (2011) propose to use eye tracking for the adaptation itself, for example, by determining when a different visualisation should be displayed if the current one is not working for the student. Mathews et al. (2012) used eye tracking to determine students’ understanding of OLM representations. Students used different OLM visualisations to answer questions requiring understanding of the representations, and the number of fixations was factored in to calculate students’ efficiency with understanding a representation. Future work also includes the possibility of presenting a different OLM visualisation if the student is struggling with the one displayed.

#### Summary

Eye tracking can be used to check whether students look at feedback (Gluck et al. 2000) or often look away from the screen and so may not concentrating (Wang et al. 2006). Eye-gaze data has also been demonstrated to improve prediction of self-explanation in comparison to using only system interaction logs (Conati and Merten 2007). Better-performing students are selective about which areas of the screen they focus on, which is particularly noticeable for more difficult problems (Kardan and Conati 2012). Experts also focussed on relevant areas more than novices (Canham and Hegarty 2010; Jarodzka et al. 2010; Kasarskis et al. 2001), and better problem solvers are able to identify irrelevant areas and shift their attention to relevant ones (Tsai et al. 2012).

Our work is differentiated from the previously discussed research, firstly, by the investigation of tutorial dialogues in the context of a constraint-based ITS and, secondly, by the ill-defined nature of the task. The research we performed can be classified under using eye-tracking data to study attention, as described in the following sections.

### Methods

As stated earlier, our study investigates how students interact with tutorial dialogues in EER-Tutor, by using student-system interaction logs as well as eye-tracking data. The first goal of the study is to identify whether there is a difference in how novices and advanced students interact with the dialogues. Of particular interest is to identify whether there are areas of the system’s interface to which a particular type of students do not pay attention. Findings of this type could be used to provide advice to students during tutorial dialogues. The second goal of the study is to investigate whether it is possible to predict future errors based on the tutorial dialogues that a student has received. Such investigation could allow for further improvements of tutorial dialogues. The findings related to these goals could enable the system to provide proactive interventions and therefore improve learning.

#### Participants

The participants were 27 Computer Science students (9 females), aged from 18 to 50 years old (mean 23.8 years, standard deviation 7.3 years). All participants had normal or corrected-to-normal vision. The participants were enrolled in a second-year database course at the University of Canterbury and volunteered to take part in the study. Each participant took part in the study individually and was given a NZ$20 voucher on completion of the study session.

#### Materials

The version of EER-Tutor used in the study excluded interface features unneeded for the study (such as scrolling). The dialogue prompts and options vary in length, so we always displayed the options in the same position to ease the definition of areas of interest. In addition, the tutorial dialogues were not adaptive. That is, when two students submit identical solutions, they would both receive the same dialogue regardless of their student model. This means that the dialogue length is only affected by the correctness of the students’ answers.

We used the Tobii TX300 eye tracker, which allows unobtrusive eye tracking as it is an integrated eye tracker. Participants are able to move during the tracking session while accuracy and precision are maintained at a sampling rate of 300 Hz (Tobii Technology 2010).

#### Procedure

The participants initially read an information sheet, signed a consent form and provided their age and vision status. A calibration phase with the eye tracker was then carried out. This involves the participant following a marker on a 9-point grid with their eyes. The participants were instructed to complete or at least attempt all of the problems and to submit their solutions regularly.

During the session, the students could work on three problems and were free to move between problems. Two problems were of moderate difficulty, and the last one was the most difficult. We selected problems that describe real-world situations the participants were familiar with (a health club, student accommodation services and the Olympic games). The students built up the diagrams incrementally and were free to choose the order in which they model elements and the elements’ positions in the solution area. Each diagram was therefore different, which differs from the majority of the related work above. Each student was given 50 min to solve the problems. Participants were reminded to regularly submit their solutions during the session. The mean session length was 49.1 min (standard deviation 3.0 min). One participant was excluded because no eye-tracking data was collected.

#### Prior knowledge groups

During the week prior to our study, the participants had a regular lab session, in which they first completed a pre-test and then used EER-Tutor. This pre-test was made up of six questions and included these question types: problem solving (drawing an EER diagram for a given scenario), multiple choice, short answer and justification. The maximum mark on the test was 27.

Following the data collection study, we classified the participants as novice or advanced using a median split on pre-test scores (median score was 13.50). This resulted in 13 novices (mean = 10.50, SD = 2.30) and 13 advanced students (mean = 16.50, SD = 1.94). Using a median split means calculating the median pre-test score for the participants and using this as the threshold for defining groups: a novice is a student with a pre-test score of 13.50 or less and an advanced student has a pre-test score greater than 13.50. Because of the small sample size, we used non-parametric statistical analysis methods. A Mann-Whitney *U* test confirms that there is a significant difference in the distributions of pre-test scores between the two groups (*U* = 0, *p* < 0.001).

### Analysing EER-Tutor logs

In this section, we present the results of the EER-Tutor log analyses: a comparison of novice and advanced students and future error prediction. There were 502 submissions (i.e. solution attempts) in total and 1285 prompts seen. Because of the small sample size, we used non-parametric statistical analysis methods. The distribution of each statistic across groups was tested using the independent-samples Mann-Whitney *U* test with ∝ = 0.05. This test is used for all novice-advanced student comparisons in this paper.

#### Analysing the behaviours of novices and advanced students

Table 1 shows a summary of the statistics for the novice and advanced students. As expected, the distributions of the mean number of completed problems are significantly different. Advanced students solved more problems on average, but the distributions of the mean number of submissions and time spent per completed problem are not significantly different. When we also consider attempted problems that were not completed, we see a significant difference in the distributions of the mean time spent per problem. Only six novices attempted the most difficult problem, while almost all advanced students worked on that problem (12 out of 13).

**Table 1:** Summary mean statistics for novice and advanced students (standard deviations reported in brackets)

	Novice	Advanced	*U* (sig.)
Completed problems	1.08 (1.12)	2.15 (0.69)	39.50 (0.019**)
Submissions per completed problem	10.29 (5.06)	11.31 (5.17)	39.50 (NS)
Time per completed problem (min)	15.08 (9.71)	16.67 (10.61)	37.00 (NS)
Time per attempted problem (min)	20.21 (3.97)	17.38 (3.72)	127.00 (0.029**)
Submissions	19.15 (8.32)	19.46 (8.26)	77.00 (NS)
Single-level dialogues seen	0.91 (0.83)	0.74 (0.49)	84.50 (NS)
Dialogues seen	15.77 (6.70)	15.08 (8.09)	86.50 (NS)
Prompts seen	53.92 (22.30)	44.92 (26.26)	106.00 (NS)
Dialogue length	3.43 (0.21)	2.99 (0.45)	158.00 (<0.001**)
Time per prompt (min)	0.28 (0.13)	0.26 (0.11)	93.00 (NS)
Unique relevant constraints	113.77 (8.88)	117.69 (3.28)	70.50 (NS)
Violated constraints	128.08 (44.51)	105.23 (83.90)	124.50 (0.039**)

**represents significance at the .05 level

Because the number of submissions, dialogues (both single- and multi-level) and prompts seen were not significantly different, we analysed finer-grained measures. The distributions of the dialogue length were significantly different. We expected this result as novices may not always answer prompts correctly because they have misconceptions or missing domain knowledge (reflected by dialogue length). The dialogue length is affected by the correctness of the students’ answers as an incorrect answer to a conceptual prompt results in a simpler conceptual prompt being given for example. Interestingly, the distributions of the time spent per prompt were not significantly different; in other words, novices and advanced students spend approximately the same time thinking about a prompt and answering it.

The distributions of the number of unique relevant constraints were not significantly different because all students were solving the same problem set. The distributions of the mean number of violated constraints were significantly different as expected however.

A comparison between the number of prompts of each type seen by novices and advanced students did not reveal any unexpected results. We expected that advanced students would make fewer incorrect choices because they began with higher prior domain knowledge; the distributions are marginally different as seen in Table 2. There was no significant difference in the percentage of help choices made by the two groups; this result suggests that novices may be unaware that they require assistance in answering prompts.

**Table 2:** A comparison of the mean percentage of choices made by novices and advanced students (standard deviations reported in brackets)

	Novice	Advanced	*U* (sig.)
Correct choices	78.13 (13.46)	85.36 (9.34)	59.00 (NS)
Help choices	2.63 (2.93)	1.95 (3.19)	104.00 (NS)
Incorrect choices	17.62 (10.75)	10.88 (8.26)	117.5 (0.10*)
Unanswered prompts	1.62 (1.55)	1.81 (2.31)	84.00 (NS)
Correct choices for reflective prompts	73.11 (16.01)	84.71 (12.59)	49.00 (0.072*)
Incorrect choices for reflective prompts	21.60 (14.10)	9.08 (8.84)	133.00 (0.012**)

*represents marginal significance at the .1 level

**represents significance at the .05 level

Because we are interested in students’ interactions with the tutorial dialogues, it is useful to look at the time spent on prompts and answer correctness for each prompt type. We had expected to find some differences in the distributions of mean prompt reflection times for the different prompt types but this was not the case. While a conceptual prompt may not require the student to read the problem statement or spend time reflecting on their solution, this is expected behaviour when interacting with a reflective prompt for example. The distributions of correct and incorrect answers for reflective prompts are marginally and significantly different, respectively (see Table 2). While these findings are not surprising, it is interesting that this is the only prompt type with differences between the two groups.

#### Predicting future errors

We investigated how the tutorial dialogues seen by the students can be used to predict future errors. That is, we want to know if a student receives a dialogue after violating constraint *C*, with what accuracy the violation (or satisfaction) of *C* can be predicted the next time it is relevant to the student’s solution. A reliable predictive system is valuable because it can be used by EER-Tutor to adapt its dialogue content or even select which violated constraint to initiate a tutorial dialogue about. In addition, after going through a dialogue about a specific constraint, violating/satisfying that constraint the next time it is relevant is a measure of the effectiveness of the dialogue.

There were 45 unique constraints discussed in the 401 multi-level dialogues seen by the 26 students. Once we discarded the dialogues corresponding to the last time a constraint was relevant during the session, we were left with 341 dialogues in the dataset. For each dialogue, we extracted six features, presented in Table 3. The first feature is the constraint identifier which is treated as a categorical feature. Including this feature allows us to have predictors (classifiers) whose prediction can vary depending on the type of constraint. The second feature is the problem number. The feature is treated as a numeric feature because its value also indicates the difficulty of the problem. The fifth feature, prior knowledge, is used to allow the possibility of predicting feature vectors from different prior knowledge groupings, separately. The last feature is the class label which will be used as the target feature for training classifiers.

**Table 3:** The dialogue data used for prediction of future errors

Feature	Description
Constraint	The id of the EER-Tutor constraint being discussed
Problem	The problem the student is working on (problems are ordered by complexity level in ascending order)
Dialogue length	The number of prompts in the dialogue
Percentage correct (PC)	The percentage of prompts the student answered correctly
Prior knowledge (PK)	Indicates whether the student is a novice or advanced student
Next occurrence	Indicates whether the constraint is violated or not the next time it is relevant to the student’s solution

We have focussed only on constructing decision trees and rule sets because the models are easy to visualise and interpret. These classifiers would also be the most straightforward to build directly into EER-Tutor. We used RapidMiner (version 5.3) for constructing classifiers (Mierswa et al. 2006). We chose the following classifiers: Decision Tree, W-J48, W-JRip and W-PART. The Decision Tree models are generated through recursive partitioning (Akthar and Hahne 2012), with all features available when selecting a feature for splitting. Reduced error pruning was enabled for the W-J48 classifier in order to generate a pruned C4.5 decision tree (Akthar and Hahne 2012; Quinlan 1993). W-JRip is a propositional rule learner that implements the RIPPER algorithm (Akthar and Hahne 2012; Cohen 1996). After each iteration, the W-PART classifier builds a partial C4.5 decision tree and turns the ‘best’ leaf into a rule (Akthar and Hahne, 2012) so we end up with a PART decision list (Frank and Witten 1998). Leave-one-out cross-validation (LOOCV) is carried out on the normalised data. LOOCV, through an iterative process, first trains a classifier and then applies it in order to measure its performance (North 2012). In each iteration, a single feature vector is used for testing, with the remaining feature vectors being used for training a classifier. The process consists of building a model using the training data and using the model to predict whether a constraint will be violated the next time it is relevant to the student’s solution. This process is repeated until each feature vector is used once for testing.

#### Prediction results

Accuracy and kappa statistic are used as measures of prediction performance. The kappa measure accounts for the possibility of the correct prediction occurring by chance (denoted by a value of 0). The highest kappa statistic value is 1, which indicates that the result is not due to chance. The kappa statistic value range is therefore usually [0,1] but negative values are possible (indicating correct predictions are occurring less than due to chance). Accuracy is calculated as the number of correct predictions over the total number of predictions. The results are shown in Table 4.

**Table 4:** Classifier performance for predicting future errors

Classifier	Accuracy (%)	Kappa
W-PART	75.66	0.236
W-J48	74.78	0.270
W-JRip	73.61	0.155
Decision tree	71.26	0.156

We obtained an accuracy of 75.66 % with a kappa statistic of 0.236 for the W-PART classifier. The W-J48 classifier has a higher kappa value (0.270) but similar accuracy (74.78 %). The kappa values for each grouping are below 0.5 for even the best-performing classifiers. This is partly affected by the fact that the kappa value tends to be stricter when there are only a few categories (here, we have two categories) (Strijbos et al. 2006).

**Fig. 4:**
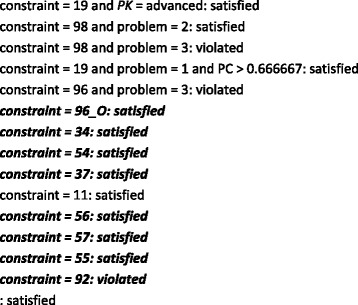
The rules generated by W-PART

Figure 4 shows the rules generated by the W-PART classifier, while the corresponding confusion matrix is given in Table 5. The first rule predicts that if the student is advanced, constraint 19 is not likely to be violated the next time it is relevant. This constraint is about the use of a regular relationship in place of another construct. There is another rule about the same constraint, specifying that students who interact with a dialogue discussing this constraint when solving the first problem and correctly answer more than two-thirds of the dialogue’s prompts are not also likely to violate it next time they use it. This suggests that novices may be overwhelmed by the complexity of the third problem, even though they may appear to understand the concept in the context of the easier problem. That is, the dialogue is more effective for the easier problem.

**Table 5:** Confusion matrix for the W-PART classifier

	True satisfied	True violated	Class precision (%)
Predicted satisfied	237	75	75.96
Predicted violated	8	21	72.41
Class recall (%)	96.73	21.88	–

Constraints 96 and 98 are only violated in subsequent attempts if the students are solving problem 3 (the most complex problem). These constraints cover the participation of entities in relationships, a concept that students new to EER data modelling often struggle with. An advanced student will not violate constraint 96 in the future even if s/he answers fewer than 80 % of the prompts correctly for problem 1. This may be an indication of slips made by the student when answering the prompts or a reflection of advanced students’ ability to retain knowledge learned from the tutorial dialogues. In the case of constraint 98, students solving the easier problem 2 do not violate the constraint next time it is relevant. A single dialogue appears to be sufficient for most of the remaining constraints as they are not violated from this point onwards. Again, this demonstrates that such students clearly understand the complex problem and therefore likely to know how to solve it. Constraint 92 is always violated in contrast, which is understandable as it covers the concept of cardinality. Even if we as domain experts perceive participation and cardinality to be of similar complexity, students appear to grasp the concept of participation more easily than that of cardinality. The effectiveness of this particular dialogue therefore needs to be investigated. One possibility is to enhance the dialogue with a worked example. In all other situations, the constraint will be satisfied. There are 69 instances in which this occurs, 19 of which are misclassified.

**Fig. 5:**
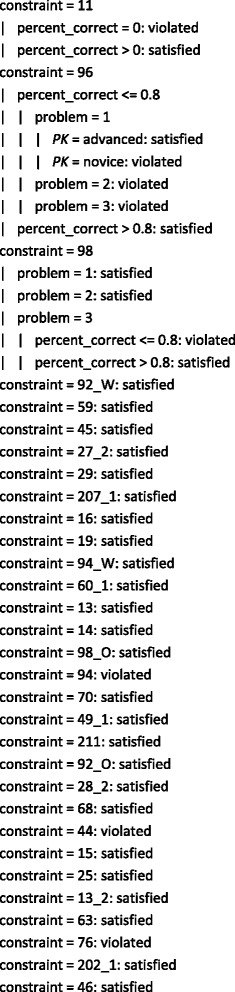
The tree generated by W-J48

The tree generated by W-J48 is shown in Fig. 5 (see Table 6 for the corresponding confusion matrix). The *highlighted* rules in Fig. 4 are also output by W-J48 but not shown in Fig. 5 due to space constraints. The percentages of correct choices made during a dialogue are the most important feature when determining the future violations of constraints 11 and 96. Constraint 11 concerns the use of a regular entity in place of another construct. This is a basic concept, and it therefore makes sense that if the student cannot answer the prompts correctly, s/he is expected to make the error again. The problem complexity and the student’s prior knowledge group also play a part for constraint 96. In contrast, future violations of constraint 98 are determined by the complexity of the problem and then the percentages of correct choices. This result is not unexpected as the students who are able to correctly answer more than 80 % of this constraint’s dialogue prompts are probably more likely to know what they are doing in subsequent attempts at the problem.

**Table 6:** Confusion matrix for the W-J48 classifier

	True satisfied	True violated	Class precision (%)
Predicted satisfied	225	66	77.32
Predicted violated	20	30	60.00
Class recall (%)	91.84	31.25	–

The confusion matrices of the two classifiers, W-PART and WJ48, are presented in Tables 5 and 6. The confusion matrices are generated based on the classification of the test data and summarise the classifier’s performance. Each confusion matrix specifies two measures, precision and recall, for each class (in our case, violated or satisfied constraints). Precision is the number of correct predictions (i.e. correctly predicted satisfied or violated constraints) as a percentage of all predicted violated or satisfied constraints. Recall is a similar metric and shows the number of correctly predicted satisfied or violated constraints as a percentage of all satisfied or violated constraints. Recall for the two classes is equivalent to *true positive rate* (TPR) and *true negative rate* (TNR).

The precision of the two classifiers on the two classes are reasonably high; that is, the majority of the predictions for either of the two classes turn out to be correct. As for recall, while most (96.73 % for W-PART and 91.84 % for J48) of the constraints that turn out to be satisfied are predicted correctly, only a small percentage (21.88 % for W-PART and 31.25 % for J48) of violated cases are identified correctly. In other words, assuming that the violation and satisfaction of a constraint correspond to a positive and negative signal correspondingly, the classifiers exhibit low *false positive* rates and high *false negative* rates. One can achieve a different trade-off between false positive and false negative by changing the relative importance of correct prediction of violation or satisfaction of a constraint through using a cost matrix when training the classifiers. If the predictor is going to be used for taking preventive actions, then, perhaps a high false negative rate (the current situation) is better than a high false positive rate as the chance of disrupting well-performing students would be lower.

### Analysing eye-tracking data

For the data during which prompts were visible, we output the following metrics from Tobii Studio:

- Fixation duration (seconds): duration of each individual fixation in an AOI.
- Fixation count: number of times the participant fixates on an AOI.

Table 7 shows the above metrics for novices and advanced students for the whole EER-Tutor interface. The distributions of the mean fixation duration are not significantly different, with a shorter mean fixation duration for advanced students. More informative results should be possible when we break down the EER-Tutor interface into key areas especially because the distributions of the mean fixation count are significantly different. Advanced students are making fewer fixations, and so it would be useful to see the differences between where novices and advanced students look.

**Table 7:** Summary mean eye-gaze metrics for novice and advanced students (standard deviations reported in brackets)

	Novice	Advanced	*U* (sig.)
Fixation duration (s)	0.26 (0.06)	0.23 (0.04)	114.50 (NS)
Fixation count	226.23 (107.09)	135.01 (68.07)	131.00 (0.016**)

**represents significance at the .05 level

**Fig. 6:**
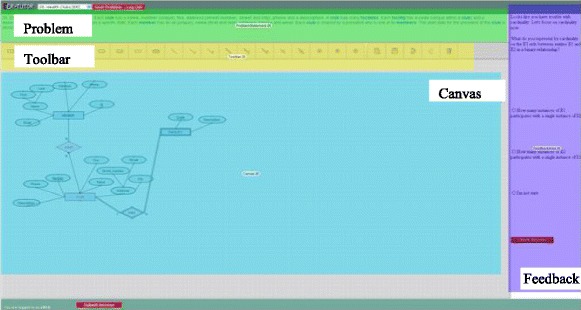
The AOIs defined for the EER-Tutor interface

The AOIs we defined for the EER-Tutor interface are shown in Fig. 6. The problem and feedback are where the problem text and dialogues are displayed, respectively. Users build their diagram on the canvas, and the toolbar has been included because it would be interesting to see if it is being used despite it being disabled during dialogues. The length of the prompt text and options varies so extra space has been included to ensure they are displayed in the same position in order to make it possible to analyse finer-grained metrics for the feedback AOI (for example transitions between the three options).

**Fig. 7:**
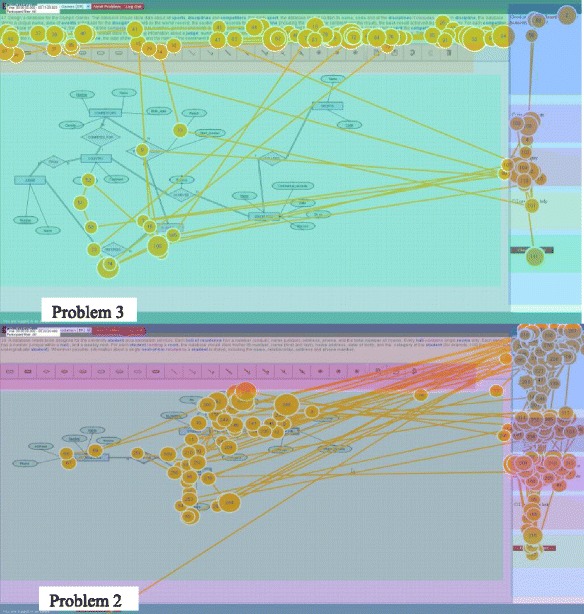
Gaze patterns for one student: corrective action prompts (*top*) and conceptual prompts (*bottom*)

Another reason to use AOIs is to make the eye-gaze patterns clearer by defining regions with specific functions. For example, Fig. 7 shows two gaze patterns of the same advanced student: the first pattern is for all corrective action prompts and the second for conceptual prompts. It is clear that the student is re-reading the problem statement for the corrective action prompts but not the conceptual prompts. This is not surprising because conceptual prompts discuss problem-independent domain knowledge while the corrective action prompt refers to the error in the diagram. It therefore follows that the student may refer back to the problem statement for corrective action prompts in order to clarify some details of the scenario.

We have not completed any complex gaze-pattern analysis to date, so Table 8 shows the analysis of the above eye-gaze metrics but breaks down the results by both the prompt type and AOI. Visit count is the number of visits to an AOI and is output from Tobii Studio. This is different to the fixation count: there can be several fixations in a single visit to an AOI. The prompt column is given to indicate the value of each metric while that specific prompt type is displayed. Only marginally and significantly different results are included.

**Table 8:** Summary mean eye-gaze metrics comparing novice and advanced students: prompt types and AOIs (standard deviations reported in brackets)

	Prompt	AOI	Novice	Advanced	*U* (sig.)
Fixation duration	CO	Canvas	0.24 (0.02)	0.20 (0.03)	134.50 (0.001**)
	CR	Canvas	0.22 (0.06)	0.17 (0.08)	112.50 (0.06*)
	CR	Problem	0.30 (0.09)	0.19 (0.05)	32.00 (0.026**)
Fixation count	CO	Canvas	70.93 (38.99)	24.32 (13.07)	141.00 (<0.001**)
	CO	Feedback	214.70 (103.18)	117.44 (87.39)	121.00 (0.019**)
	CR	Canvas	21.46 (19.73)	8.64 (11.54)	122.00 (0.016**)
	CR	Problem	8.92 (6.09)	3.08 (1.96)	29.00 (0.093*)
Visit count	CO	Canvas	17.45 (9.06)	7.49 (5.18)	138.00 (0.001**)
	CO	Feedback	30.32 (21.01)	17.18 (17.65)	113.50 (0.052*)
	CA	Feedback	5.96 (3.12)	4.08 (1.70)	86.50 (0.08*)
	CR	Canvas	5.73 (4.07)	3.91 (4.23)	110.50 (0.077*)
	CR	Feedback	18.61 (16.95)	11.45(7.94)	90.00 (0.052*)

*represents marginal significance at the .1 level

**represents significance at the .05 level

Conceptual and conceptual reinforcement prompts are problem-independent, and so there is no need to look at the canvas or problem statement to answer the prompts. The distributions of the mean fixation durations for the canvas AOI are different for these two prompt types however. For conceptual reinforcement prompts, there is also a significant difference in the distributions of the mean fixation durations for the problem AOI. The reason for both groups looking at the canvas may be the fact that the error is highlighted in red on the diagram and so is eye-catching. The difference may be that advanced students do not look at their solutions for as long because they do not need it to answer the prompt. This is supported by the advanced students’ lower visit counts to the canvas AOI but further investigation is needed. Novices therefore fail to identify that these prompts are problem-independent, possibly because they are unable to generalise the error and retain knowledge for the future. In addition, the dialogues provide feedback, and students naturally assume that they need to relate the dialogue content with the diagram.

The distributions of the mean fixation counts are significantly different for the canvas and feedback AOIs of conceptual prompts. The canvas and problem AOIs’ distributions are also significantly different for conceptual reinforcement prompts. Again, there is no reason to look at the canvas other than to look at the highlighted error as discussed above. While advanced students have a lower mean fixation count on the feedback area, it may be that they are able to select the correct option more quickly than novices.

It is interesting that there are no significant differences between novices and advanced students for reflective prompts as seen in Table 8. More details of the students’ behaviour would be revealed by breaking down the feedback AOI into prompt text and individual option AOIs.

**Fig. 8:**
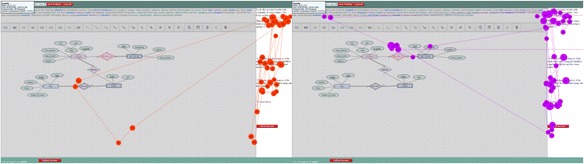
The gaze patterns of the same prompt for an advanced (*left*) and novice (*right*) student

The distributions of the mean visit counts are marginally different for the feedback AOI for corrective action, conceptual reinforcement and conceptual prompts. This can be a result of advanced students making fewer transitions between the AOIs because they recognise the relevant AOIs for each prompt type and focus on those (in this case, the feedback area), whereas novices are unaware of the relevance of the different areas and therefore more frequently switched between AOIs. In fact, when looking at the same conceptual prompt for the same problem for an advanced and a novice student shows that the overall pattern may appear similar initially (see Fig. 8). After considering the order of the fixations, however, it becomes clear that the advanced student looks at the canvas only once before returning and focussing on the feedback AOI for the rest of the time the prompt is displayed. This is in contrast to the novice, who switches between the feedback and canvas more frequently and even looks at the problem statement briefly. Further investigation of the transitions is required however as this is a quick observation of behaviour of these two specific students.

It should be noted that the values for novices are higher than for advanced students for all metrics reported in Tables 7 and 8. Similar to Kasarskis et al. (2001), we found that advanced students have shorter fixation durations on average. It was clear that advanced students are more aware of which areas are irrelevant for problem-independent prompts for example. While more detailed analysis is required regarding gaze transition patterns, related work suggests similar findings (see the ‘Related eye-tracking research’ section).

## Conclusions and future work

We presented the preliminary results of a study that investigated students’ interactions with tutorial dialogues in EER-Tutor. Both eye-gaze and student-system interaction logs were used as data sources. The ultimate aim is to use one or both data sources to allow EER-Tutor to further support students by detecting sub-optimal behaviours and adapting its behaviour to students.

It is evident that there are some differences between novices and advanced students in terms of their behaviour as indicated by the collected EER-Tutor and eye-tracking data. From the EER-Tutor logs, we see that there is no significant difference in the distributions of the percentage of help choices made by the two groups. Novices therefore are not aware of situations in which they need help. A way to deal with this would be to enable the ITS to intervene and explain the error being discussed in more detail. Time-based evidence from EER-Tutor logs, like the time spent on a prompt, does not show any differences between novices and advanced students. This suggests a gap in our knowledge about students’ behaviour and another data source such as eye-gaze data can be combined with the EER-Tutor log data so that we better understand students. The eye-gaze data reveal that advanced students are more selective about the areas they focus on and make fewer visits to irrelevant AOIs. While this needs to be investigated further, it demonstrates that it should be possible for an ITS to eventually detect sub-optimal behaviours that produce these effects from both sources in real-time and react appropriately. An example of sub-optimal behaviour is a student who does not refer to his/her solution at all when interacting with the tutorial dialogues. The ITS can intervene in such situations, suggesting to the student that it is beneficial to inspect the solution.

We predicted whether a specific constraint would be violated on its next occurrence with an accuracy of over 74 %. This information can be used to further improve the ITS to support students who do not learn from dialogues. For example, the ITS can adapt or enhance the dialogue content, maybe by giving an example of the same error in a different context and explaining why it is wrong and how to correct it. We found that students had difficulty with the participation concept. This is a complex concept that requires a lot of knowledge about entities and relationships as well as a deep understanding of the problem scenario. Students therefore only satisfy this constraint if they have sufficient knowledge and can answer prompts correctly.

We intend to analyse the collected data further, in particular by investigating the transitions between the different AOIs of the EER-Tutor interface (the students’ gaze patterns) and using machine learning to discover commonly occurring learning behaviours. One of the next steps is to build classifiers that are able to automatically place students into the appropriate group in real time. Classifiers built using only EER-Tutor data, only eye-gaze data and both data sources will be compared. This will eliminate the need for the pre-test scores we used to categorise students. The performance of these classifiers will help us determine whether the cost of eye tracking is justified: features calculated from EER-Tutor may provide reasonably accurate student classification that is relatively inexpensive to collect. Possible features that we can use to classify students include the number of correctly solved problems and the number of transitions between the different AOIs. This work also needs to be incorporated into EER-Tutor to provide adaptive interventions and guidance, which themselves are directions for future research. We also plan to perform similar research with other ITSs (teaching difference instructional tasks) to test the generality of our findings.

## References

[CR1] Akthar F, Hahne C (2012). RapidMiner 5 Operator Reference.

[CR2] Aleven V, Koedinger KR, Cross K, Lajoie SP, Vivet M (1999). Tutoring answer explanation fosters learning with understanding. Proceedings of the 9th International Conference on Artificial Intelligence in Education, AIED ’99.

[CR3] Aleven V, Ogan A, Popescu O, Torrey C, Koedinger K, Lester JC, Vicari RM, Paraguaçu F (2004). Evaluating the effectiveness of a tutorial dialogue system for self-explanation. Proceedings of Seventh International Conference on Intelligent Tutoring Systems.

[CR4] Bull S, Cooke N, Mabbott A, Conati C, McCoy K, Paliouras G (2007). Visual attention in open learner model presentations: An eye-tracking investigation. User Modeling 2007.

[CR5] Bunt A, Conati C, Huggett M, Muldner K (2001). On improving the effectiveness of open learning environments through tailored support for exploration. Proceedings of AIED 2001, 10th World Conference of Artificial Intelligence and Education.

[CR6] Canham M, Hegarty M (2010). Effects of knowledge and display design on comprehension of complex graphics. Learning and Instruction.

[CR7] Chi MTH (2000). Self-explaining expository texts: the dual process of generating inferences and repairing metal models. Advances in Instructional Psychology.

[CR8] Chi MTH, Bassok MM, Lewis MW, Reimann P, Glaser R (1989). Self-explanations: how students study and use examples in learning to solve problems. Cognitive Science.

[CR9] Cohen WW (1996). Proceedings of the Thirteenth National Conference on Artificial Intelligence (AAAI-96).

[CR10] Conati C, Merten C (2007). Eye-tracking for user modeling in exploratory learning environments: An empirical evaluation. Knowledge-Based Systems.

[CR11] Conati C, Carenini G, Harati M, Tocker D, Fitzgerald N, Flagg A (2011). User-adaptive visualizations: can gaze data tell us when a user needs them?. Proceedings of Scalable Integration of Analytics and Visualization (Vol. 2).

[CR12] Eivazi S, Bednarik R (2011). Predicting problem-solving behavior and performance levels from visual attention data. 2nd Workshop on eye gaze in intelligent human machine interaction.

[CR13] Elmasri R, Navathe SB (2007). Fundamentals of database systems.

[CR14] Frank E, Witten I (1998). Generating accurate rule sets without global optimization. Proceedings of the 15th International Conference on Machine Learning (pp. 144–151). Morgan Kaufmann.

[CR15] Gluck KA, Anderson JR, Douglass SA, Gauthier G, Frasson C, VanLehn K (2000). Broader bandwidth in student modeling: What if ITS Were “Eye”TS?. ITS ’00: Proceedings of the 5th International Conference on Intelligent Tutoring Systems.

[CR16] Goldberg JH, Helfman JI (2010). Comparing information graphics: a critical look at eye tracking. Proceedings of the 3rd BELIV’10 Workshop: BEyond time and errors: novel evaLuation methods for Information Visualization.

[CR17] Graesser AC, Jackson GT, Mathews EC, Mitchell HH, Olney A, Ventura M, Alterman R, Kirsh D, Group T.R (2003). Why/AutoTutor: A test of learning gains from a physics tutor with natural language dialog. Proceedings of the twenty- fifth annual conference of the cognitive science society.

[CR18] Jarodzka H, Scheiter K, Gerjets P, van Gog T (2010). In the eyes of the beholder: How experts and novices interpret dynamic stimuli. Learning and Instruction.

[CR19] Kardan S, Conati C (2012). Exploring gaze data for determining user learning with an interactive simulation. User Modeling, Adaptation, and Personalization.

[CR20] Kasarskis P, Stehwien J, Hickox J, Aretz A, Wickens C, Jensen R (2001). Comparison of expert and novice scan behaviors during VFR flight. Proceedings of the 11th International Symposium on Aviation Psychology.

[CR21] Koedinger KR, Anderson JR (1998). Illustrating principled design: The early evolution of a cognitive tutor for algebra symbolization. Interactive Learning Environments.

[CR22] Law, B, Atkins, MS, Kirkpatrick, AE, Lomax, AJ, & Mackenzie, CL. (2004). Eye Gaze Patterns Differentiate Novice and Experts in a Virtual Laparoscopic Surgery Training Environment. In A T Duchowski & R Vertegaal (Eds.), *Proceedings of the 2004 symposium on Eye tracking research & applications* (pp. 41–48).

[CR23] Mathews M, Mitrovic A, Lin B, Holland J, Churcher N, Cerri S, Clancey W, Papadourakis G, Panourgia K (2012). Do your eyes give it away? Using eye tracking data to understand students’ attitudes towards open student model representations. Intelligent Tutoring Systems.

[CR24] Mierswa I, Wurst M, Klinkenberg R, Scholz M, Euler T, Ungar L, Craven M, Gunopulos D, Eliassi-Rad T (2006). Yale: Rapid prototyping for complex data mining tasks. Proceedings of the 12th ACM SIGKDD International Conference on Knowledge Discovery and Data Mining.

[CR25] North DMA (2012). Data mining for the masses (p. 264). Global Text Project.

[CR26] Olney AM, Graesser AC, Person NK, Nkambou R, Bourdeau J, Mizoguchi R (2010). Tutorial dialog in natural language. Advances in intelligent tutoring systems (pp. 181–206) Springer.

[CR27] Quinlan JR (1993). C4.5 programs for machine learning. Morgan Kaufmann.

[CR28] Strijbos J-W, Martens RL, Prins FJ, Jochems WMG (2006). Content analysis: What are they talking about?. Computers & Education.

[CR29] Suraweera P, Mitrovic A (2004). An intelligent tutoring system for entity relationship modelling. International Journal of Artificial Intelligence in Education.

[CR30] Tobii Technology AB (2010). Tobii TX300 Eye Tracker Product Description.

[CR31] Tsai M-J, Hou H-T, Lai M-L, Liu W-Y, Yang F-Y (2012). Visual attention for solving multiple-choice science problem: An eye-tracking analysis. Computers & Education.

[CR32] Vanlehn K, Jordan PW, Rosé CP, Bhembe D, Böttner M, Gaydos A, Srivastava R, Cerri SA, Gouardères G, Paraguaçu F (2002). The architecture of Why2-Atlas: A coach for qualitative physics essay writing. Proceedings of the 6th International Conference on Intelligent Tutoring Systems.

[CR33] Wang H, Chignell M, Ishizuka M (2006). Empathic tutoring software agents using real-time eye tracking. Proceedings of the 2006 symposium on Eye tracking research & applications - ETRA ’06.

[CR34] Weerasinge A, Mitrovic A (2005). Supporting deep learning in an open-ended domain. Studies in fuzziness and soft computing.

[CR35] Weerasinghe A, Mitrovic A (2006). Facilitating deep learning through self-explanation in an open-ended domain. International Journal of Knowledge-Based and Intelligent Engineering Systems (KES).

[CR36] Weerasinghe A, Mitrovic A, Martin B (2008). A preliminary study of a general model for supporting tutorial dialogues. 16th International Conference on Computers in Education.

[CR37] Weerasinghe A, Mitrovic A, Martin B (2009). Towards individualized dialogue support for ill-defined domains. International Journal of Artificial Intelligence in Education.

[CR38] Weerasinghe A, Mitrovic A, Thomson D, Mogin P, Martin B, Biswas G, Bull S, Kay J, Mitrovic A (2011). Evaluating a general model of adaptive tutorial dialogues. Proceedings of the 15th international conference on Artificial intelligence in education.

[CR39] Zakharov K, Mitrovic A, Ohlsson S, Looi C-K, McCalla G, Bredeweg B, Breuker J (2005). Feedback micro-engineering in EER-Tutor. Artificial intelligence in education.

